# The role of health literacy in the association between academic performance and substance use

**DOI:** 10.1093/eurpub/ckab213

**Published:** 2022-01-05

**Authors:** Jaana M Kinnunen, Leena Paakkari, Arja H Rimpelä, Markus Kulmala, Matthias Richter, Mirte A G Kuipers, Anton E Kunst, Pirjo L Lindfors

**Affiliations:** 1 Unit of Health Sciences, Faculty of Social Sciences, Tampere University, Tampere, Finland; 2 Research Centre for Health Promotion, Faculty of Sport and Health Sciences, University of Jyväskylä, Jyväskylä, Finland; 3 Department of Adolescent Psychiatry, Pitkäniemi Hospital, Nokia, Tampere University Hospital, Tampere, Finland; 4 Institute of Medical Sociology, Medical Faculty, Martin Luther University Halle-Wittenberg, Halle (Saale), Germany; 5 Department of Public and Occupational Health, Amsterdam Public Health Research Institute, Amsterdam UMC, University of Amsterdam, Amsterdam, The Netherlands

## Abstract

**Background:**

To address social inequalities in adolescent substance use and consequent disparities in health, it is important to identify the mechanisms of the association between substance use and academic performance. We study the role of health literacy (HL) in the association between academic performance and weekly smoking, monthly alcohol use and cannabis ever-use among adolescents in Europe.

**Methods:**

SILNE-R school survey data, which was collected in 2016–17 with paper-and-pencil-method from Hanover (GE), Amersfoort (NL) and Tampere (FI), were used (*N* = 5088, age 13–19). Health Literacy for School-aged Children instrument was used to assess students’ HL. Logistic regression analyzed the association of substance use with academic performance and HL, separately and in the same model. Linear and multinomial logistic regression analyzed the association between academic performance and HL.

**Results:**

Poor academic performance compared with high was associated with smoking [odds ratio (OR) 3.94, 95% confidence interval (CI) 2.83–5.49], alcohol use (OR: 2.94, 95% CI: 2.34–3.68) and cannabis use (OR: 2.56, 95% CI: 1.89–3.48). Poor HL was also associated with each substance use (with ORs of 2.32, 1.85 and 1.29). HL was positively associated with academic performance (β = 1.04, 95% CI: 0.89–1.20). The associations between academic performance and substance use were only slightly attenuated after controlling for HL.

**Conclusions:**

Academic performance and HL were both determinants of substance use, confirming their role in tackling the disparities in substance use. However, HL did not demonstrably mediate the association between academic performance and substance use. A wider set of factors needs to be tackled to address emerging social inequalities in adolescent substance use.

## Introduction

Adolescence is the time when health-compromising behaviours, like substance use, are adopted.^1^ These behaviours may have far-reaching influences on later health in adulthood and play a part in the development of social disparities in health.^2–5^ To tackle the disparities, it is important to identify the underlying mechanisms that explain social patterning in these behaviours.

Academic performance has been recognized as a significant social stratifier related to differences in substance use.^4^^,^^6–12^ Longitudinal studies have proven that the association between academic performance and substance use is reciprocal and thus mutually reinforcing; low academic performance not only predicts more substance use but also is affected by the use.^4^^,^^6^^,^^12^ An example of causal mechanisms from academic performance to substance use could be that adolescents from low socioeconomic status families more often have lower school performance compared to adolescents from high socioeconomic status families,^13^ and they more often have lower psychosocial resources within the family, e.g. a divorced family and poor contacts with the parents, and educational and peer environments.^14^ Family socioeconomic position, which reflects on adolescents’ academic performance, may influence substance use through these psychosocial characteristics.^15^

To better understand the mechanisms explaining the association between academic performance and substance use, it is important to study possible mediating factors influencing the association. One such factor may be health literacy (HL). HL has been acknowledged as a key pillar of public health and health promotion strategies and actions in efforts to address health disparities.^16^ According to WHO,^16^ among other skills, HL allows people to ‘make informed health decisions and lifestyle choices, assess health information and understand health messages in the public domain’ (Ref. 16, pp. 4–5), which may be crucial in interpreting health warning messages and choosing not to engage in substance use. As HL is positively associated with academic performance,^17^^,^^18^ this may explain the social gradient in substance use.

HL has been found to be related to smoking or intention to smoke^17^^,^^19^^,^^20^ and alcohol use^17^^,^^19^^,^^21^ among school-aged children. A systematic review^22^ showed that 13 out of 17 studies had found significant linear relationships between HL and adolescent health behaviours. A longitudinal study found that lower HL at baseline was associated with a greater increase in substance use later.^23^ It has been suggested that part of the association between academic performance and smoking and alcohol use may be mediated by HL.^17^^,^^24^ If HL plays an important role in substance use disparities, it would be an important leverage point in tackling disparities in substance use that are linked to academic performance. However, earlier studies about the mediating role of HL between academic performance and substance use have been national-level studies and focused mainly on smoking and alcohol use.^17^^,^^24^ Not much is known about whether the mediating role is similar in different countries, or in relation to cannabis.

**Figure 1:**
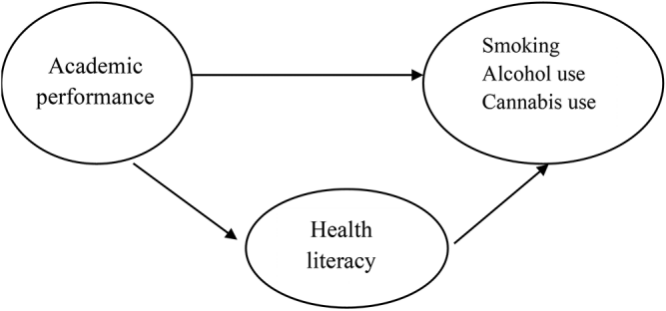
A theoretical pathway model explaining the associations between academic performance, health literacy and substance use (smoking, cannabis and alcohol)

The aim of this study is to explore HL’s role in the association between academic performance and substance use among adolescents with data collected from different settings, i.e. three cities in three European countries. More specific research questions are as follows: (i) What kind of association is there between academic performance and substance use (i.e. tobacco, alcohol and cannabis)? (ii) How is HL associated with substance use? (iii) Does HL mediate the association between academic performance and substance use (figure 1)? These will be analyzed first with all data and then stratified by city to check whether the results are similar for each city.

## Methods

### Participants and study procedure

The data used in this study were collected during the academic year 2016–17 as part of the SILNE-R (Enhancing the effectiveness of programs and strategies to prevent youth smoking: a comparative realist evaluation of 7 European cities) school survey in three European cities (country): Amersfoort (the Netherlands; 6 schools), Hanover (Germany; 12 schools) and Tampere (Finland, 9 schools), as the questions on HL were included in the survey only in these cities. The cities were selected on the basis of their population size, income and employment rate to be close to the national average, and different types of schools were invited to participate. Two school grades were selected in each country to cover 14- to 16-year-old students from different socioeconomic backgrounds and with different levels of academic performance. All students in these grades were invited to participate (*N* = 6453), which led to an age range from 12 to 19, due to variation in birthdays and ages when starting school. The survey was conducted with paper-and-pencil method during regular school hours. Instantly after completion, the questionnaires were sealed in envelopes and the answers were entered into a web platform by the responsible organization of each country. The overall response rate was 78.8% (*N* = 5088); in Amersfoort (NL), it was 84.9%, in Hanover (GE) 65.8% and in Tampere (FI) 87.1%. The study protocol was approved by appropriate ethical committees in all the survey countries. More information on the SILNE-survey can be found in Lorant et al.^25^

### Measures

Health literacy was assessed with the Health Literacy for School-aged Children (HLSAC) instrument, which contains 10 items covering five components: theoretical knowledge, practical knowledge, critical thinking, self-awareness and citizenship.^26^ The Cronbach’s alpha for the scale was 0.912; in Amersfoort (NL) it was 0.895, in Hanover (GE) 0.921 and in Tampere (FI) 0.917. A sum variable was calculated for those with none or one missing item, and three categories were created according to the instrument:^26^ high (36–40 points), average (26–35 points) and low (10–25 points). Those with more than one missing item (4.0%) were excluded as most of them had all ten items missing (2.7%).

Academic performance was assessed by asking ‘Which of the following best describes your school marks during the past year?’ The measurement instrument was adjusted for each country’s grading system, but it separated students into five categories: high, good, average, low and insufficient. In the analyses, high and good categories were combined as ‘Good performance’, and low and insufficient categories were combined as ‘Low performance’ while average performance remained as such.

Alcohol use was asked with a question: ‘Thinking back over the last 12 months, how often did you have a drink of alcohol (more than just a sip)?’ Respondents were dichotomized into drinking at least monthly (‘Yes’) or drinking less than monthly, which also included non-drinkers (‘No’).

Smoking was based on two questions: ‘Have you ever tried cigarette smoking, even just a few puffs?’ with options ‘No’ and ‘Yes’ and ‘How many cigarettes have you smoked during the last 30 days?’ The responses were dichotomized according to weekly smoking as ‘No’, also including non-smokers, and ‘Yes’ (smokes at least one cigarette per week).

Cannabis use was assessed with a question: ‘Thinking back over the last 12 months, how often did you use marijuana or cannabis (a joint, pot, weed, hash…)?’ The responses were dichotomized as ‘No’ (has never tried, which was the first answering option) and ‘Yes’ (has used during the last 12 months or earlier, i.e. lifetime use).

Control variables in the analyses were age (range: 12–19 years), gender, immigrant background and parental education. Parental educational level was used as a proxy for parental socioeconomic status, and it was asked for the father and the mother separately. The question was adapted for each country. For the analyses, the answers were combined as the highest educational level of either parent. A common four-category variable was used: high, middle, low and do not know/other. Immigrant background was assessed with the question, separately for mother and father: ‘In which country was your mother/father born?’ If at least one of the parents was born in some other country than the survey country, the respondent was classified as having an immigrant background.

### Data analysis

First, a series of descriptive statistics were performed with all data and for each city separately. To test statistical differences, the Pearson χ^2^ test was used. The mediation was analyzed with series of regression analyses. The association between academic performance and HL was analyzed first with linear regression analysis and then with categorized variables with multinomial logistic regression analysis. To analyze whether academic performance and HL were associated with monthly alcohol use, weekly smoking and cannabis ever-use, binary logistic regression analyses were conducted for academic performance and HL separately, with control variables. Finally, a multivariate logistic regression analysis was conducted with academic performance, HL, substance use (for monthly alcohol use, weekly smoking and cannabis ever-use separately) and control variables in the same model. The Wald χ^2^ test was used to test for statistical significance of the explanatory variables. All the analyses were conducted first for all data and then stratified by city. An analysis of variance test was used to test the variance between schools in HL. School explained 7% of the variance for HL, so the logistic regressions were analyzed with generalized linear mixed models, which take school clustering into account. IBM SPSS Statistics, V.26, was used for all these data analyses.

Additionally, a path analysis was conducted to study the relationship of academic performance and HL in predicting substance use with the software language R, version 4.0.5, and the package lavaan, an R Package for Structural Equation Modeling. Analysis was first conducted for all data and then separately for each city. For each model, age, gender, immigrant status and parents' educational level were controlled for.

## Results

Table 1 presents the characteristics of the study populations overall and stratified by city. Health literacy was the highest in Tampere (FI), then in Amersfoort (NL) and the lowest in Hanover (GE) both in group mean points and when categorized (*P* < 0.001). Weekly smoking, monthly alcohol use and cannabis ever-use were more frequent in Amersfoort (NL) than in Hanover (GE) and Tampere (FI).

**Table 1:** Descriptive statistics of the study population, all and by city (*n* = number of participants)

Variable	All (*n* = 5088)	Amersfoort (NL) (*n* = 1858)	Hanover (GE) (*n* = 1497)	Tampere (FI) (*n* = 1733)
Mean age, years (SD)	14.65 (0.87)	15.00 (0.85)	14.20 (0.90)	14.67 (0.68)
Age, range, years	12–19	13–18	12–19	13–17
Gender, %				
Boys	51.6	52.8	50.0	51.8
Girls	48.4	47.2	50.0	48.2
Health literacy [mean (SD)], %	32.55 (5.29)	32.85 (4.96)	31.28 (5.43)	33.30 (5.32)
High	30.9	31.0	21.1	39.2
Average	61.4	63.8	68.7	52.7
Low	7.7	5.2	10.2	8.1
Academic performance, %				
Good	35.6	30.1	39.9	38.1
Average	45.0	53.9	47.4	33.5
Low	19.3	16.0	12.7	28.4
Monthly alcohol use, %				
No	79.8	68.0	82.8	90.0
Yes	20.2	32.0	17.2	10.0
Weekly smoking, %				
No	93.0	90.5	95.1	93.8
Yes	7.0	9.5	4.9	6.2
Cannabis ever-use, %				
No	91.3	86.1	91.6	96.6
Yes	8.7	13.9	8.4	3.4
Highest parental education, %				
High	48.5	54.6	54.0	37.1
Average	26.4	19.1	25.3	35.2
Low	5.7	10.3	4.3	1.9
Unknown	19.4	16.0	16.4	25.8
Immigrant background, %				
No	74.6	78.7	57.6	85.0
Yes	25.4	21.3	42.4	15.0

### Health literacy and academic performance

Both with linear regression and multinomial logistic regression, a statistically significant positive association was found between academic performance and HL (table 2). With linear regression, HL increased 1.04 points with each level of academic performance (95% CI: 0.89–1.20). With multinomial logistic regression, the strongest association was found between low academic performance and low HL when compared to high categories (OR: 4.05; 95% CI: 2.86–5.76). When stratified by cities, this association was the strongest in Tampere (FI): OR 8.09 with 95% CI of 4.33–15.10 (Supplementary table S1). The regression coefficient for academic performance in predicting HL was 1.123 for all data, 1.808 for Tampere (FI), 1.456 for Hanover (GE) and 0.259 for Amersfoort (NL).

**Table 2:** Adjusted ORs^a^ and 95% CIs from bivariate multinomial logistic regression for average and low HL compared to high HL by academic performance, and linear regression between academic performance and HL

Logistic regression			Linear regression		
Variable	Contrast in HLS	OR (95% CI)	β	(95% CI)	P
Academic performance (ref: high)			1.04	0.89–1.20	<0.001
Average	Average vs. high	1.39 (1.20–1.61)			
	Low vs. high	1.87 (1.38–2.54)			
Low	Average vs. high	1.92 (1.57–2.34)			
	Low vs. high	4.05 (2.86–5.76)			
*P*^b^		<0.001			

a Adjusted for age, gender, parental education, immigrant background and school clustering.

b Wald χ^2^ test to test for statistical significance of the explanatory variables in the model.

### Academic performance, HL and substance use

The adjusted associations for substance use (smoking, alcohol and cannabis use) are presented in table 3. The results of Model 1 (academic performance and HL analyzed separately) show that both academic performance and HL were associated with substance use, but academic performance somewhat more strongly than HL. For all substances, the associations with academic performance hardly attenuated after controlling for HL, e.g. for weekly smoking, the OR of low academic performance was 3.94 (95% CI: 2.83–5.49) in Model 1, and in Model 2, it was 3.72 (2.65–5.23). Conversely, the associations with HL substantially weakened after controlling for academic performance. For weekly smoking, e.g. the OR of low academic performance was 2.32 (1.56–3.45) in Model 1, while it was 1.81 (1.21–2.73) in Model 2 (table 3). The interaction term of academic performance and HL, which was added in Model 2, was not statistically significant for weekly smoking (*P* = 0.401), for monthly alcohol use (*P* = 0.281), nor for cannabis ever-use (*P* = 0.376).

**Table 3:** Adjusted ORs^a^ and the 95% CIs from logistic regression for substance use by academic performance and health literacy and the *P* values^b^ for statistical significance of the fixed effect of the variable in the model

Variable	Model 1	Model 2
Weekly smoking		
Academic performance		
High	1.00	1.00
Average	1.52 (1.11–2.08)	1.43 (1.04–1.98)
Low	3.94 (2.83–5.49)	3.72 (2.65–5.23)
*P*^b^	<0.001	<0.001
Health literacy		
Good	1.00	1.00
Average	1.19 (0.90–1.56)	1.07 (0.80–1.41)
Low	2.32 (1.56–3.45)	1.81 (1.21–2.73)
*P*^b^	<0.001	0.009
Monthly alcohol use		
Academic performance		
High	1.00	1.00
Average	1.75 (1.45–2.11)	1.71 (1.41–2.07)
Low	2.94 (2.34–3.68)	2.88 (2.28–3.63)
*P*^b^	<0.001	<0.001
Health literacy		
Good	1.00	1.00
Average	1.32 (1.10–1.57)	1.22 (1.02–1.46)
Low	1.85 (1.37–2.50)	1.56 (1.14–2.12)
*P*^b^	<0.001	0.012
Cannabis ever-use		
Academic performance		
High	1.00	1.00
Average	1.28 (0.97–1.67)	1.23 (0.94–1.62)
Low	2.56 (1.89–3.48)	2.38 (1.74–3.25)
*P*^b^	<0.001	<0.001
Health literacy		
Good	1.00	1.00
Average	1.23 (0.96–1.57)	1.18 (0.92–1.51)
Low	1.29 (0.84–1.98)	1.11 (0.72–1.74)
*P*^b^	0.222	0.446

Notes: Model 1: Academic performance and health literacy separately, controlled for age, gender, immigrant background, parental education and school clustering. Model 2: All variables from model 1 simultaneously in the same model.

a Adjusted for age, gender, parental education, immigrant background, and school clustering.

b Wald χ^2^ test to test for statistical significance of the explanatory variables in the model.

The path analysis showed that both HL and academic performance were significant factors in predicting substance use, as better HL and academic performance predicted a lower probability for substance use. The regression coefficient was −0.370 for academic performance in predicting weekly smoking, −0.288 in predicting monthly alcohol use and −0.291 in predicting cannabis ever-use. The regression coefficient was −0.021 for HL in predicting weekly smoking, −0.017 in predicting monthly alcohol use and 0.039 in predicting cannabis ever-use.

Supplementary table S2 shows the adjusted multivariate associations of academic performance, HL and substance use stratified by city. The strongest association in Model 1 was found in Tampere (FI) between low academic performance and weekly smoking (OR: 10.65 with 95% CI: 5.23–21.68) and the weakest in Hanover (GE) between low HL and cannabis ever-use (OR: 0.90 with 95% CI: 0.41–1.96). HL was not statistically significantly associated with any substance use in Hanover (GE), with weekly smoking and cannabis ever-use in Amersfoort (NL), or with cannabis ever-use in Tampere (FI). Academic performance was not statistically significantly associated with weekly smoking in Hanover (GE). Generally, the multivariate results for specific cities also showed that the relationships with academic performance persisted, while the relationships with HL weakened, after mutual control. (Supplementary table S2).

## Discussion

### Key findings

We aimed to study HL’s role in the association between academic performance and substance use (tobacco, alcohol and cannabis). We found that academic performance was positively associated with HL. Both academic performance and HL were associated with substance use, i.e. low academic performance and low HL were associated with a higher prevalence of weekly smoking, monthly alcohol use and cannabis ever-use, so both HL and academic performance are significant factors in predicting substance use. Generally, our results suggest that academic performance is a stronger determinant for substance use than HL. Notably, HL does not demonstrably contribute to the association between academic performance and substance use. This was also found in city-wise analyses, but there were differences between cities in the associations.

### Interpretation of the findings

Our results on the associations between academic performance and HL, and academic performance and HL and substance use, could mean that academic performance and HL are both independent determinants of substance use. However, academic performance was more strongly associated with substance use than HL was. The association between academic performance and substance use has been confirmed in many studies.^4^^,^^6–12^

When comparing our results on the association between HL and substance use to previous results, there is a slight contradiction. It is notable that the HL instrument has not been the same in all other studies. Paakkari et al.^17^ found HL as a mediator between academic performance and smoking and alcohol use, but we did not find a clear indication of mediation. However, the statistical analyses were not the same, with also some differences in key questions, the reason why the comparison is not airtight. Beside our study, Rüegg and Abel^24^ found no empirical support for an effect of HL on smoking among 18- to 25-year-old male adults in Switzerland, but the methods were different from ours. Additionally, there was no statistically significant association in our study between HL and any substance use in Hanover (GE). This might be due to a notably larger proportion of average and low HL in Hanover (GE). In Amersfoort (NL), statistically significant association with HL was found only for monthly alcohol use, and in Tampere (FI) for weekly smoking and monthly alcohol use. However, a systematic review^22^ has confirmed the associations between HL and adolescent health behaviours. Furthermore, as previous studies using a nationally representative sample in Finland have confirmed that enhancing adolescents’ HL may help in preventing substance use at the national level, our findings using city-level samples may indicate that within a country, region-specific interventions may be needed.

Mazanov and Byrne’s longitudinal study^27^ revealed that smokers’ factual knowledge on the health consequences of smoking were better compared to non-smokers’ knowledge. Good factual knowledge may be unrelated to actual smoking behaviour and therefore not straightforwardly helpful in prevention, as there is vast evidence that combined social competence or social influences curricula in smoking prevention have significant long-term effects in the school context.^28^ Interventions should also target a comprehensive set of competencies, such as adolescents’ decision-making and judgment skills on health issues which are not yet fully developed, as suggested also by Fleary et al.,^22^ as merely theoretical or factual knowledge may not prevent substance use.

Adolescents’ academic performance reflects their parents’ socioeconomic position^13^ and psychosocial resources within the family.^14^ Parents’ socioeconomic position is also associated with adolescents’ health-compromising behaviours.^4^ Additionally, parental education, income and adult health literature are related to adolescent HL.^22^ This may indicate that parents with higher education, income and HL can offer better resources and other opportunities to their children to learn and practice these skills further, as suggested by Fleary et al.^22^ Given that studies focusing on the role of families on adolescents’ HL development are rare, more research is needed on the issue to make further conclusions. Thus, all these factors, i.e. family background, adolescent academic performance and HL, are interrelated and associated with substance use, which creates a challenge for studies. To tackle these challenges, longitudinal studies with data on students, their families and school would be needed.

In our study, we found differences in HL between countries: it was the highest in Tampere (FI), then in Amersfoort (NL) and the lowest in Hanover (GE). This is in line with the earlier findings that have showed differences between the countries among adolescents.^26^^,^^29^ This may be due to a difference in how teaching of health topics is organized in schools. While in the Netherlands and Germany health education is integrated into other school subjects, such as biology, and the intensity of curricula varies between schools, in Finland, it is an obligatory and independent school subject, ‘Health Education’ (HE), for grades 7–9 in basic education and in upper secondary education. For grades 1–6, HE is taught as part of environmental studies. In the Finnish national core curriculum, HL is used as a theoretical framework for defining and describing the goals for learning in HE.^30^ The subject is organized around key themes such as growth and development; health in everyday choices; resources and coping skills; and health, society and culture.^31^ Additionally, the HE teachers, like other teachers in Finland, are well educated; the teacher’s qualification means a master’s degree. So, HL could be thought of more as an academic skill in Finland. This assumption is also supported by our result on academic performance and HL, which were positively associated, and this association was the strongest in Tampere (FI).

### Strengths and limitations

We used self-reported data and cannot know how accurately the students answered the items of the HLSAC instrument or how well their perceived competence in HL echoes their actual competence. However, the role of perceived competence in explaining health behaviour has been widely acknowledged.^32^ The HLSAC instrument was also the last question in the questionnaire, which may have increased the number of missings and number of students being too tired to read the items properly, which might have weakened the associations. However, <5% were missing in HLSAC items. The low response rate in Hanover (GE) was due to active consent, and thus, its data may be biased and may not entirely represent the target population; in Amersfoort (NL) and Tampere (FI), passive consents were used. The quite small Ns for low HL and cannabis ever-use groups, especially in stratified analyses, could have widened the confidence intervals. Cannabis ever-use is rare among adolescents of this age, and we used the cut-off points for different categories given for the HLSAC instrument.^26^ The instrument is a cross-national measurement, and our study population had generally quite high HL levels. Although the cities selected for this study were quite average ones according to sociodemographic factors,^25^ the cities may not represent the whole country in their HL levels. Thus, these results may not be generalized to the national context.

Despite these limitations, this study has many strengths. The same survey and HL instrument were used, and the same study procedure was followed in every city, which enabled comparisons between cities. The sample was large, which allowed stratified analyses in different cities. We also included cannabis use in our study, which has been rarely studied in the context of academic performance and HL.

## Conclusions

In our study, both low HL and low academic performance were associated with substance use, which confirms their role in tackling the disparities in substance use. However, HL did not demonstrably mediate the association between academic performance and substance use. Enhancing students’ HL may help in substance use prevention and to some extent in reducing inequalities in substance use. A wider set of factors needs to be tackled to address emerging academic performance-related social inequalities in substance use in adolescence. In addition, adolescents’ HL needs to be studied more with nationally representative samples and longitudinally to explore its potential role in substance use.

## Supplementary data

Supplementary data are available at *EURPUB* online.

## Supplementary Material

ckab213_Supplementary_Data
