# Supplementary material for: The role of health literacy in the association between academic performance and substance use
Source: Eur J Public Health. 2022 Jan 5;32(2):182–7. doi: 10.1093/eurpub/ckab213 (PMC8975541; doi:10.1093/eurpub/ckab213)
Supplement: ckab213_Supplementary_Data [file ckab213_supplementary_data.zip › ckab213-suppl_data/ejph-2021-06-om-0758-File003.pdf]

Supplementary Table 1. Adjusted odds ratios[¥] (OR) and 95% confidence intervals (CI) from bivariate multinomial logistic regression for average and low HL compared to high HL by academic performance, and linear regression between academic performance and HL, by city.

**Logistic regression**		**OR (95% CI)**		
**Variable**	**Contrast in HL**	**Amersfoort (NL)**	**Hanover (GE)**	**Tampere (FI)**
**Academic performance (ref: High)**				
Average	Average vs high	1.21 (0.96–1.53)	1.49 (1.12–1.99)	1.42 (1.11–1.82)
	Low vs high	1.04 (0.61–1.79)	2.11 (1.30–3.44)	3.10 (1.66–5.77)
Low	Average vs high	1.33 (0.96–1.85)	2.50 (1.44–4.33)	2.13 (1.58–2.88)
	Low vs high	2.23 (1.15–4.32)	3.75 (1.78–7.89)	8.09 (4.33–15.10)
*p**		.044	.001	< .001
**Linear regression**		**β (95% CI)**		
**Variable**		**Amersfoort (NL)**	**Hanover (GE)**	**Tampere (FI)**
**Academic performance**		0.130 (-0.166–0.426)	1.22 (0.88–1.57)	1.53 (1.31–1.74)

[¥] Adjusted for age, gender, parental education, immigrant background, and school clustering

* Wald χ^2 test to test for statistical significance of the explanatory variables in the model

HL = health literacy
