# Supplementary material for: The role of health literacy in the association between academic performance and substance use
Source: Eur J Public Health. 2022 Jan 5;32(2):182–7. doi: 10.1093/eurpub/ckab213 (PMC8975541; doi:10.1093/eurpub/ckab213)
Supplement: ckab213_Supplementary_Data [file ckab213_supplementary_data.zip › ckab213-suppl_data/ejph-2021-06-om-0758-File004.pdf]

Supplementary Table 2. Adjusted odds ratios[¥] (OR) and the 95% confidence interval (CI) from logistic regression for weekly smoking, monthly alcohol use and cannabis ever-use in each city by academic performance and health literacy (HL) and the p-value* for statistical significance of the fixed effect of the variable in the model.

	**Amersfoort (NL)**		**Hanover (GE)**		**Tampere (FI)**	
	Model 1	Model 2	Model 1	Model 2	Model 1	Model 2
Weekly smoking						
**Academic performance**						
Good	1.00	1.00	1.00	1.00	1.00	1.00
Average	1.25 (0.83–1.90)	1.26 (0.83–1.91)	1.22 (0.72–2.06)	1.11 (0.64–1.90)	2.06 (0.96–4.45)	1.92 (0.85–4.36)
Low	2.68 (1.66–4.34)	2.54 (1.56–4.14)	1.95 (1.01–3.75)	1.80 (0.91–3.56)	10.65 (5.23–21.68)	10.94 (5.15–23.25)
p^*	< .001	< .001	.129	.206	< .001	< .001
**Health literacy**						
High	1.00	1.00	1.00	1.00	1.00	1.00
Average	1.18 (0.81–1.71)	1.13 (0.79–1.65)	1.29 (0.68–2.44)	1.22 (0.63–2.34)	0.95 (0.58–1.56)	0.76 (0.45–1.27)
Low	1.89 (0.99–3.63)	1.72 (0.89–3.31)	2.11 (0.94–4.73)	1.70 (0.72–4.02)	2.63 (1.39–4.97)	1.65 (0.85–3.19)
p^*	.159	.273	.176	.478	.002	.038

Monthly alcohol use						
**Academic performance**						
Good	1.00	1.00	1.00	1.00	1.00	1.00
Average	1.66 (1.28–2.17)	1.69 (1.29–2.22)	1.73 (1.25–2.41)	1.67 (1.20–2.34)	1.87 (1.20–2.91)	1.73 (1.10–2.73)
Low	3.30 (2.35–4.63)	3.22 (2.28–4.55)	1.76 (1.10–2.83)	1.79 (1.09–2.89)	3.93 (2.50–6.17)	3.77 (2.36–6.02)
p^*	< .001	< .001	.003	.006	< .001	< .001
**Health literacy**						
High	1.00	1.00	1.00	1.00	1.00	1.00
Average	1.37 (1.08–1.75)	1.34 (1.04–1.72)	1.18 (0.82–1.70)	1.08 (0.75–1.57)	1.22 (0.85–1.75)	1.06 (0.73–1.52)
Low	1.88 (1.14–3.10)	1.73 (1.04–2.89)	1.10 (0.64–1.92)	0.96 (0.54–1.70)	2.68 (1.58–4.54)	1.85 (1.08–3.19)
p^*	.010	.028	.669	.842	.001	.060
Cannabis ever-use						
**Academic performance**						
Good	1.00	1.00	1.00	1.00	1.00	1.00
Average	0.98 (0.69–1.38)	0.96 (0.67–1.37)	1.85 (1.14–3.00)	1.82 (1.11–2.98)	1.11 (0.60–2.06)	1.06 (0.56–1.99)
Low	1.75 (1.15–2.66)	1.67 (1.09–2.56)	3.22 (1.75–5.93)	2.73 (1.43–5.19)	2.60 (1.43–4.73)	2.51 (1.36–4.65)
p^*	.004	.010	.001	.007	.001	.002

**Health literacy**						
High	1.00	1.00	1.00	1.00	1.00	1.00
Average	1.04 (0.76–1.42)	1.01 (0.74–1.39)	1.38 (0.82–2.34)	1.35 (0.78–2.33)	1.25 (0.76–2.05)	1.16 (0.70–1.92)
Low	1.55 (0.82–2.94)	1.43 (0.75–2.73)	0.90 (0.41–1.96)	0.70 (0.30–1.62)	1.38 (0.60–3.15)	1.12 (0.48–2.58)
p^*	.393	.526	.271	.152	.616	.842

Model 1: Academic performance and HL separately, controlled for age, gender, immigrant background, parental education, and school clustering

Model 2: All variables from Model 1 simultaneously in the same model

[¥] Adjusted for age, gender, parental education, immigrant background, and school clustering

* Wald χ^2 test to test for statistical significance of the explanatory variables in the model
